# Efficient Fourier base fitting on masked or incomplete structured data

**DOI:** 10.3389/fnimg.2025.1480807

**Published:** 2025-06-04

**Authors:** Fariba Karimi, Esra Neufeld, Arya Fallahi, Vartan Kurtcuoglu, Niels Kuster

**Affiliations:** ^1^The Foundation for Research on Information Technologies in Society (IT'IS), Zurich, Switzerland; ^2^Department of Information Technology and Electrical Engineering, Swiss Federal Institute of Technology (ETH), Zurich, Switzerland; ^3^The Interface Group, Institute of Physiology, University of Zurich, Zurich, Switzerland

**Keywords:** Fourier-base fitting, image processing, reconstruction, brain deformation data, masked data

## Abstract

**Introduction:**

Fourier base fitting for masked or incomplete structured data holds significant importance, for example in biomedical image data processing. However, data incompleteness destroys the simple unitary form of the Fourier transformation, necessitating the construction and solving of a linear system—a task that can suffer from poor conditioning and be computationally expensive. Despite its importance, suitable methodology addressing this challenge is not readily available.

**Methods:**

In this study, we propose an efficient and fast Fourier base fitting method suitable for handling masked or incomplete structured data. The developed method can be used for processing multi-dimensional data, including smoothing and intra-/extrapolation, even when confronted with missing data.

**Results:**

The developed method was verified using 1D, 2D, and 3D benchmarks. Its application is demonstrated in the reconstruction of noisy and partially unreliable brain pulsation data in the context of the development of a biomarker for non-invasive craniospinal compliance monitoring and neurological disease diagnostics.

**Discussion:**

The study investigated the impact of different analytical and numerical performance improvement measures (e.g., term rearrangement, precomputation of recurring functions, vectorization) on computational complexity and speed. Quantitative evaluations on these benchmarks demonstrated that peak reconstruction errors in masked regions remained acceptable (i.e., below 10 % of the data range for all investigated benchmarks), while the proposed computational optimizations reduced matrix assembly time from 843 s to 11 s in 3D cases, demonstrating a 75-fold speed-up compared to unoptimized implementations. Singular value decomposition (SVD) can optionally be employed as part of the solving-step to provide regularization when needed. However, SVD quickly becomes the performance limiting in terms of computational complexity and resource cost, as the number of considered Fourier modes increases.

## 1 Introduction

Fast Fourier transform (FFT) is an efficient algorithm that transforms a signal from its original domain (usually time or space) to the frequency domain. In particular, it is used to compute the discrete Fourier transform (DFT) or its inverse (IDFT) of a signal. FFT is extensively employed in a wide range of applications such as filtering, efficient convolution evaluation, wave propagation, and more. It can be interpreted as a special basis transformation to the orthonormal Fourier basis $v_{k}\left(r\right)=e^{jk\cdot r}/C$ ($j=\sqrt{-1}$, *C*: normalization constant). When the spatial domain consists of a structured rectangular grid with *L*_*i*_ (typically an even number) equidistant coordinates along the *i*-axis with extent *l*_*i*_, ***k*** is equal to (*n*_*x*_·2π/*l*_*x*_, *n*_*y*_·2π/*l*_*y*_, *n*_*z*_·2π/*l*_*z*_) with −*L*_*i*_/2 ≤ *n*_*i*_ < *L*_*i*_/2. While the DFT is defined for regular grids and the FFT is applicable to data in that structured form, there is often a need to fit a Fourier basis to unstructured data, or to structured data known on a subset of the grid points (referred to here as “masked" data). Packages like PyNUFFT (Lin, 2018) are designed to handle unstructured data. However, PyNUFFT's application to masked structured data (a special case of unstructured data) poses challenges, since PyNUFFT treats missing data as zero. This means that this package is not suited for extrapolating data based on its frequency domain representation, a common application in medical imaging.

One notable advantage of the Fourier basis lies in the simplicity of its application and similarity between the DFT and IDFT. They link the Fourier transform $\tilde{f}\left(k\right)$ to the underlying data *f*(***r***) through $\tilde{f}\left(k\right)={\text{Σ}}_{r}v_{k}^{*}\left(r\right)f\left(r\right)$ and $f\left(k\right)={\text{Σ}}_{r}v_{k}\left(r\right)\tilde{f}\left(r\right)$, where ^*^ denotes the complex conjugate. However, once the grid on which data is available seizes to be regular and complete, *v*_***k***_ stops being an orthogonal basis. In other words, ${\text{Σ}}_{r}v_{k}^{*}\left(r\right)v_{k^{\prime }}\left(r\right)$ can be non-zero, even when ***k*** ≠ ***k*****′**. In fact, merely computing ${\text{Σ}}_{r}v_{k}^{*}\left(r\right)f\left(r\right)$ no longer corresponds to determining the coordinates of *f* in the space spanned by the Fourier basis *v*, as the coordinate transformation matrix {*v*_***k***_(***r***)}***r***, ***k*** is non-unitary and often non-square. Instead, (pseudo-)inversion of said matrix is required to establish the coordinate transformation.

It is often desirable to reduce the considered Fourier base vectors to the range [−*N*_*i*_, *N*_*i*_] where *N*_*i*_ < *L*_*i*_/2. Reducing the range of considered base vectors acts as a form of low-pass filtering, effectively smoothing the signal. Fitting such a reduced basis can be valuable, e.g., for obtaining a fitted approximation for inter- or extrapolation purposes.

In the course of our investigation into establishing a non-invasive surrogate for craniospinal compliance (CC) by measuring changes in head impedance during the cardiac cycle (Karimi et al., 2023; Spiegelberg et al., 2022), we were confronted with this issue. CC is a measure of intracranial volume buffering capacity. A drop in CC, as it may occur in pathological conditions such hydrocephalus (Balédent et al., 2004), syringomyelia (Leung et al., 2016), Chiari I malformation (Terem et al., 2018), and cerebral small vessel disease (Perosa et al., 2022), often precedes a potentially life-threatening increase in intracranial pressure. Since direct continuous monitoring of CC is currently not possible, an indirect quantification with a surrogate derived from non-invasive brain motion assessment could play an important role in the clinical management of such conditions. Consequently, the quantification and interpretation of brain's pulsatile motion not only contributes to understanding brain physiology, but can also play a role in the diagnosis and therapeutic decision-making for the above-mentioned neurological disorders.

To maximize the signal information content of a potential novel CC surrogate, we leveraged magnetic resonance imaging (MRI) deformation data, illustrating how the brain pulsates during the cardiac cycle due to the exchange of blood and cerebrospinal fluid (CSF) between cranial and spinal compartments (Karimi et al., 2023). Deformation imaging is typically performed using Displacement Encoding with Stimulated Echoes (DENSE) (Soellinger et al., 2009; Adams et al., 2020; Karimi et al., 2023) or phase-contrast MRI (Enzmann and Pelc, 1992). Fourier basis fitting of the MRI data is essential for two distinct reasons (Karimi et al., 2023): first, it improves signal-to-noise ratio, which is of utmost importance considering the sensitivity of the surrogate signal prediction, the noisy nature of imaging data, and the subtlety of the features of interest. Second, Fourier basis fitting enables extrapolation of the data from the brain bulk volume. This is necessary because the displacements of primary relevance are located on the brain surface, where artifacts associated with flowing CSF and the high contrast to bone and air cavities make the displacement data unreliable, such that it has to be masked out. Similar challenges are common in the field of medical imaging, and beyond. The equivalent for masked structured data to the “FFT"-method should be fast and efficient both computationally and in terms of memory requirements, especially for processing large amounts of (e.g., 4D image) data. Since masking often results in an ill-conditioned Fourier decomposition matrix ([*A*] in Equation 8), the required inversion can also become computationally challenging.

This work has three main goals:

- Develop a fast and robust Fourier base fitting method for masked structured data;
- Verify the method using 1D, 2D, and 3D benchmarks;
- Demonstrate its applicability to deformation data in order to support computation and interpretation of head impedance change over the cardiac cycle, utilizing the approach established in Karimi et al. (2023).

## 2 Methodology

### 2.1 Linear system derivation

Let $\hat{f}\left(r\right)$ be an approximation for *f*(***r***) expressed in its Fourier basis as:


(1) $$\begin{array}{l} \hat{f}\left(r\right)=\overset{N_{z}}{\underset{n_{z}=-N_{z}}{\sum }}\overset{N_{y}}{\underset{n_{y}=-N_{y}}{\sum }}\overset{N_{x}}{\underset{n_{x}=-N_{x}}{\sum }}\\ \;\left\{a_{n_{x},n_{y},n_{z}}e^{j\left(n_{x}k_{x_{0}}x+n_{y}k_{y_{0}}y+n_{z}k_{z_{0}}z\right)}\right\}\\ \;=\underset{n}{\sum }a_{n}e^{jk_{n}\cdot r} \end{array}$$

where *N*_*i*_ are the desired number of harmonics along the *i*-axis-direction, *k*_*i*_0__ = 2π/*l*_*i*_, and *a*_*n*_ are complex numbers. If the number of spatial points are larger than the number of coefficients, the system is overdetermined and *a*_*n*_ should be computed by minimizing the difference between *f*(***r***) and $\hat{f}\left(r\right)$ using (for instance) the least square approach:


(2) $$\begin{array}{l} a_{n}=\underset{a_{n}}{\arg\min}||f\left(r\right)-\hat{f}\left(r\right){||}^{2} \end{array}$$

This can result in complex-valued $\hat{f}\left(r\right)$. Therefore, to ensure a real-valued approximation, Equation 1 is modified as follows:


(3) $$\begin{array}{l} \hat{f}\left(r\right)=\underset{n}{\sum }\left(a_{n}e^{jk_{n}\cdot r}+a_{n}^{*}e^{-jk_{n}\cdot r}\right) \end{array}$$

Defining *R* as:


(4) $$\begin{array}{l} R=\underset{r}{\sum }{\left(f\left(r\right)-\underset{n}{\sum }\left(a_{n}e^{jk_{n}\cdot r}+a_{n}^{*}e^{-jk_{n}\cdot r}\right)\right)}^{2} \end{array}$$

where the first summation is over all available points in 3D space, and expressing *a*_*n*_ in terms of real values *a*_*n*_ = *b*_*n*_+*jc*_*n*_, we obtain the following conditions by ensuring a zero gradient at minimum:


(5) $$\begin{array}{l} \begin{array}{c} \frac{\partial R\left(r\right)}{\partial b_{m}}=\underset{r}{\sum }\left(f\left(r\right)-\underset{n}{\sum }\left[a_{n}e^{jk_{n}\cdot r}+a_{n}^{*}e^{-jk_{n}\cdot r}\right]\right)\\ \times \left(e^{jk_{m}\cdot r}+e^{-jk_{m}\cdot r}\right)=0 \end{array} \end{array}$$

(6) $$\begin{array}{l} \begin{array}{c} \frac{\partial R\left(r\right)}{\partial c_{m}}=\underset{r}{\sum }\left(f\left(r\right)-\underset{n}{\sum }\left[a_{n}e^{jk_{n}\cdot r}+a_{n}^{*}e^{-jk_{n}\cdot r}\right]\right)\\ \times \left(e^{jk_{m}\cdot r}-e^{-jk_{m}\cdot r}\right)=0 \end{array} \end{array}$$

Multiplying Equations 5 and 6 by 0.5 and summing them yields:


(7) $$\begin{array}{l} \underset{r}{\sum }\left(e^{jk_{m}\cdot r}\underset{n}{\sum }\left(a_{n}e^{jk_{n}\cdot r}+a_{n}^{*}e^{-jk_{n}\cdot r}\right)\right)=\\ \underset{r}{\sum }f\left(r\right)e^{jk_{m}\cdot r} \end{array}$$

In real-valued matrix-form, Equation 7 can be written as:


(8) $$\begin{array}{l} \begin{array}{c} \left(\begin{array}{cccc} \vdots & \vdots & \ddots & \vdots \\ \cdots & Re\left\{S_{m+n}+S_{m-n}\right\} & -Im\left\{S_{m+n}-S_{m-n}\right\} & \cdots \\ \cdots & Im\left\{S_{m+n}+S_{m-n}\right\} & Re\left\{S_{m+n}-S_{m-n}\right\} & \cdots \\ \vdots & \vdots & \ddots & \vdots \end{array}\right)\\ \left(\begin{array}{c} \vdots \\ b_{n}\\ c_{n}\\ \vdots \end{array}\right)=\left(\begin{array}{c} \vdots \\ Re\left\{\underset{r}{\sum }f\left(r\right)e^{jk_{m}\cdot r}\right\}\\ Im\left\{\underset{r}{\sum }f\left(r\right)e^{jk_{m}\cdot r}\right\}\\ \vdots \end{array}\right)\equiv \left[A\right]\left[x\right]=\left[B\right] \end{array} \end{array}$$

where $S_{m+n}=\sum_{r}e^{j(k_{m}+k_{n})\cdot r}$ and $S_{m-n}=\sum_{r}e^{j(k_{m}-k_{n})\cdot r}$. In this system of equations, odd (respectively, even) rows express equations for *b*_*n*_ (*c*_*n*_). Even though it may seem that the number of unknown variables is *N* = 2(2*N*_*x*_ + 1)(2*N*_*y*_ + 1)(2*N*_*z*_ + 1), these are actually pairs of duplicates, as *b*_*n*_*x*_, *n*_*y*_, *n*_*z*__ = *b*_−_*n*__*x*_, −*n*_*y*_, −*n*_*z*__ and *c*_*n*_*x*_, *n*_*y*_, *n*_*z*__ = −*c*_−_*n*__*x*_, −*n*_*y*_, −*n*_*z*__. This arises from the fact that $\hat{f}\left(r\right)$ should be real, necessitating $a_{k}=a_{-k}^{*}$. In other words, a reduced system of equations [*AA*] and [*BB*] can be constructed from [*A*] and [*B*], such that the dimensions are halved and (2*N*_*x*_ + 1)(2*N*_*y*_ + 1)(2*N*_*z*_ + 1) unknown variables ([*xx*]) are apparent. [*x*] is recovered from [*xx*] as follows (notation: *a*:*b* signifies integers from *a* to *b*; *a*:*c*:*b* signifies the same, but in steps of *c*):


(9) $$\begin{array}{l} x[i]=\frac{1}{2}xx[i]\;i=1:\frac{N}{2}-1\\ \;x[i]=xx[i]\;i=\frac{N}{2}\\ x[N-i]=\frac{1}{2}xx[i]\;i=1:2:\frac{N}{2}-1\;\\ x[N-i+1]=-\frac{1}{2}xx[i+1]\;i=1:2:\frac{N}{2}-1 \end{array}$$

### 2.2 Efficient matrix computation

In the previous section, we obtained a system of linear equations, whose solution provides Fourier base fitting. In this section, we focus on the efficient computation of [*AA*] and [*BB*] in terms of both computational and memory resource requirements. Key considerations are the reduction of the number of function evaluations—particularly of expensive non-linear function evaluations, which are factored out, combined, and precomputed—and the enabling of vectorization. Let *M*(*x, y, z*) be the 3D mask which has a value of one where data exist and zero elsewhere. We pre-evaluate the costly exponential functions $e^{jn_{x}k_{x_{0}}x_{1D}}$, $e^{jn_{y}k_{y_{0}}y_{1D}}$, and $e^{jn_{z}k_{z_{0}}z_{1D}}$ for *n*_*x*_ = −2*N*_*x*_:2*N*_*x*_, *n*_*y*_ = −2*N*_*y*_:2*N*_*y*_, and *n*_*z*_ = −2*N*_*z*_:2*N*_*z*_, where *x*_1*D*_, *y*_1*D*_, and *z*_1*D*_ are the one dimensional *x*, *y*, and *z* coordinates. By this, we do not need to compute any other exponential in later stages. Subsequently, we compute *S*_*i*_ as follows:


(10) $$\begin{array}{l} S_{i}=\underset{r}{\sum }e^{jk_{i}\cdot r}\\ \;=\underset{x_{1D}}{\sum }\underset{y_{1D}}{\sum }\underset{z_{1D}}{\sum }M\left(x,y,z\right)e^{jk_{z_{i}}z}e^{jk_{y_{i}}y}e^{jk_{x_{i}}x}\\ \;=\underset{x_{1D}}{\sum }\underset{y_{1D}}{\sum }\left(\underset{z_{1D}}{\sum }M\left(x,y,z\right)e^{jk_{z_{i}}z}\right)e^{jk_{y_{i}}y}e^{jk_{x_{i}}x}\\ \;=\underset{x_{1D}}{\sum }\left(\underset{y_{1D}}{\sum }M_{z}\left(x,y\right)e^{jk_{y_{i}}y}\right)e^{jk_{x_{i}}x}\\ \;=\underset{x_{1D}}{\sum }M_{y,z}\left(x\right)e^{jk_{x_{i}}x} \end{array}$$

Similarly for the computation of [*BB*] matrix elements:


(11) $$\begin{array}{l} BB\left[i\right]=\underset{r}{\sum }f\left(x,y,z\right)e^{jk_{i}\cdot r}\\ =\underset{x_{1D}}{\sum }\underset{y_{1D}}{\sum }\underset{z_{1D}}{\sum }f\left(x,y,z\right)M\left(x,y,z\right)e^{jk_{z_{i}}z}e^{jk_{y_{i}}y}e^{jk_{x_{i}}x}\\ =\underset{x_{1D}}{\sum }\underset{y_{1D}}{\sum }F_{z}\left(x,y\right)e^{jk_{y_{i}}y}e^{jk_{x_{i}}x}\\ =\underset{x_{1D}}{\sum }F_{y,z}\left(x\right)e^{jk_{x_{i}}x} \end{array}$$

Note that Equations 10 and 11 take the form of a regular Fourier transformation, such that an FFT method could be employed. However, given that *N*_*i*_≪*L*_*i*_ in our application-of-interest, it is preferable to use vectorized *tensordot* operations instead (see Section 4.1). Additionally, if 4*N*_*i*_>*L*_*i*_, the number of required *S* computations can be reduced by considering *S*_*n*_ = *S*_*n* + *p*_, where *p* = (*j*_*x*_·*L*_*x*_, *j*_*y*_·*L*_*y*_, *j*_*z*_·*L*_*z*_)—in other words, exploiting symmetry modulo *L*_*i*_.

The code was implemented in Python 3.9 using the NumPy library. Pre-storage of required exponentials in 1D space and reducing 3D transformations to 1D ones (in Equations 10 and 11) results in important performance improvements in terms of computational complexity and memory requirements (see Section 4.1). Nested loops were avoided completely and *for*-loops were replaced whenever possible by vectorization and array operation. A custom least-square solving method was implemented to avoid the large memory overhead of commonly available Python implementations. This is necessary, as the first dimension of the [*A*] matrix is equal to the number of spatial points, which can be very large for volumetric image data.

### 2.3 Solving and regularization

**Figure 1:**
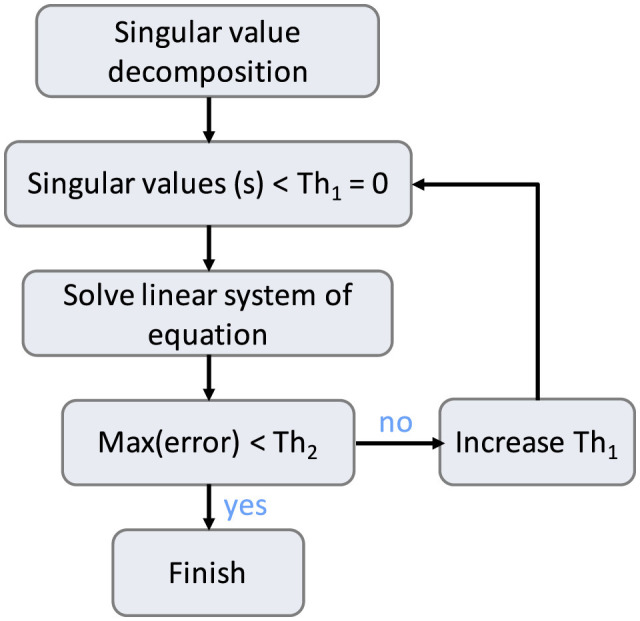
Regularization pipeline using singular value decomposition.

In the previous section, we obtained a linear system of equations ([*AA*][*xx*] = [*BB*]) that needs to be solved to obtain the correct Fourier basis. As [*AA*] is frequently ill-conditioned, we used regularization based on singular value decomposition (SVD). Figure 1 shows the regularization pipeline: first, the SVD is obtained using the NumPy linear algebra package, i. e., [*AA*] = *USV*^⊤^ where *U* and *V* are unitary matrices and *S* is diagonal. Subsequently, all singular values smaller than an adaptively adjusted threshold (see below) are zeroed in *S*. The regularized inverse of [*AA*] is obtained as ${\left[AA\right]}_{reg}^{-1}=VS^{'-1}U^{⊤}$, where *S*′ is the thresholded singular value matrix, and its inverse is easily obtained by inverting the diagonal elements. Subsequently, [*xx*] is computed using $\left[xx\right]={\left[AA\right]}_{\text{reg}}^{-1}\left[BB\right]$. To identify the singular value threshold, the maximum of relative error between $\hat{\left[BB\right]}=\left[AA\right]\left[xx\right]$ and [*BB*] is computed. If it is larger than a user-specified acceptance criterion, the number of non-zero singular values is increased by a fixed factor (we used 1.02 in the applications below) and the procedure repeated.

Note that it is only necessary to compute S and AA, and to perform the SVD and matrix inversion once for a dataset with a constant mask, even if data is gathered at multiple time-steps.

### 2.4 Reconstruction from Fourier basis coefficients

For reconstruction, a variant of Equation 3 is used:


(12) $$\begin{array}{l} \hat{f}\left(r\right)=2Re\left\{\underset{n}{\sum }\left(a_{n}e^{jk_{n}\cdot r}\right)\right\} \end{array}$$

A summary of the main computational steps of the proposed method, including references to key equations and corresponding sections, is provided in Table 1.

**Table 1:** Summary of the main computational steps in the proposed method, with references to key equations and sections.

**Step**	**Description**	**Key equations/ section**
1	Fix the number of desired Fourier modes	Section 2.1 and Section 4.2
2	Precompute recurring exponential terms	Section 2.2
3	Assemble the (reduced) linear system matrix [*AA*] and right-hand side vector [*BB*] using the rearrangement and vectorization scheme.	Equations 8–17, Section 2.1
4	Solve the resulting linear system [*AA*][*xx*] = [*BB*] either directly or via SVD-based regularization with adaptive thresholding.	Figure 1, Section 2.3
5	Reconstruct the spatial field from the estimated Fourier coefficients.	Equation 18, Section 2.4

## 3 Results

The developed method was tested on a Windows system with AMD Ryzen 9 7950X 16-Core processor and 128 GB of memory.

### 3.1 Verification benchmarks

To verify the proposed method, we defined three benchmark problems spanning from one to three spatial dimensions based on the Ackley function (normalized to a [0, 1] range), which is a common test function for global optimization methods. The full hardware configuration and implementation hyperparameters used for all benchmarks are summarized in Table 2.

**Table 2:** Implementation details and hyperparameter settings.

**Category**	**Parameter**	**Value/description**
**Hardware**	CPU	AMD Ryzen 9 7950X 16-Core processor
	GPU	NVIDIA GeForce RTX 3060
	RAM	128 GB
	OS	Windows 10
	Environment	Python 3.9, NumPy 1.26, JAX 0.4.20 (for SVD)
**Fourier fitting**	Number of Fourier modes (*N*_*x*_, *N*_*y*_, *N*_*z*_)	*N*_*i*_ = 11 in each spatial direction for all benchmarks and *N*_*i*_ = 4 for deformation data
	Benchmark function parameters	*a* = 5, *b* = 0.2, *c* = 1.5π (Ackley-type function)
	Grid resolution	200 (1D), 200 × 200 (2D), 200 × 200 × 200 (3D)
	Padding	10 %
	Masking pattern	Structured block-wise masks (see Figures 2–8)
**Regularization**	Initial singular value truncation threshold	Singular values below 0.1
	Error-based refinement criterion	Triggered if max|[*AA*·coef−*BB*]|>3 × 10^−3^
	Adjustment strategy	Increase number of retained singular values by 2% until error criterion is satisfied

#### 3.1.1 1D benchmark

**Figure 2:**
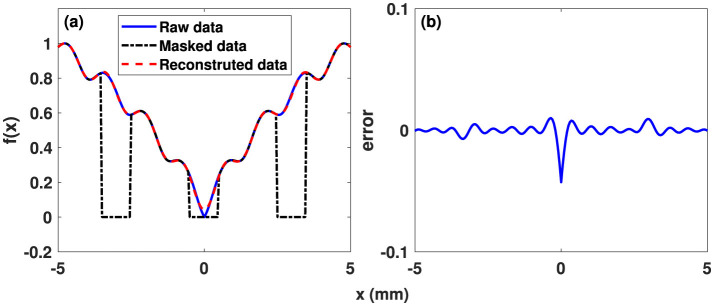
**(a)** Target function, masked raw data, and Fourier reconstructed data (*N*_*x*_ = 11). **(b)** Reconstruction deviations for the 1D benchmark (Equation 13 with *a* = 5, *b* = 0.2, *c* = 1.5π).

**Figure 3:**
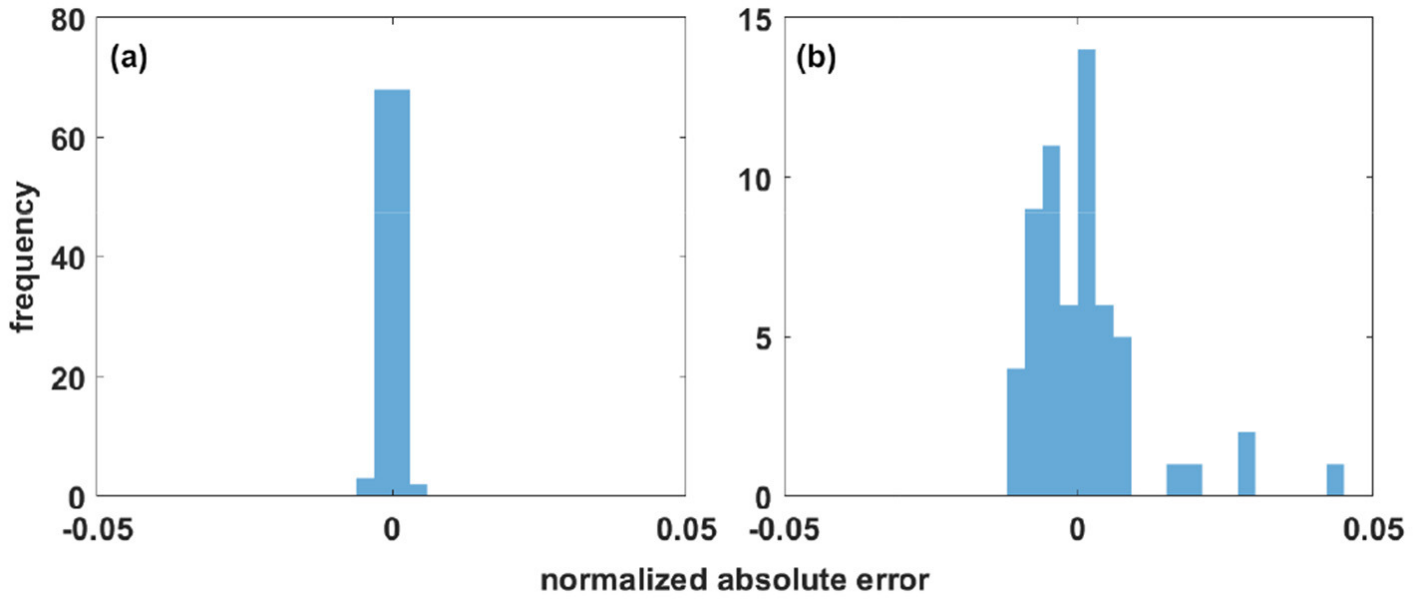
Absolute difference between the target function and the Fourier reconstructed data for the 1D benchmark presented in Equation 13. **(a)** Distribution of deviations within the mask. **(b)** Deviations in the reconstructed regions, where no data were available (“holes”). Note: The value range is 1.

Let *f*_1*D*_(*x*) be defined as follows:


(13) $$\begin{array}{l} f_{1D}\left(x\right)=\\ -a\exp\left(-b|x|\right)-\exp\left(\frac{1}{a}\cos\left(cx\right)\right)+a+\exp\left(1\right) \end{array}$$

on *x*:[−5, 5]. The interval is discretized into 200 points and it is assumed that no data is available for *x*:[−3.5, −2.5]∪[−0.5, 0.5]∪[2.5, 3.5]. The reconstruction results (parameters: *a* = 5, *b* = 0.2, *c* = 1.5π, *N*_*x*_ = 11) are shown in Figure 2. Figure 3 displays the histogram of the reconstruction error for the domains inside and outside the mask. The quality metrics and compute times are specified in Table 3, which includes results for the 2D and 3D benchmarks as well.

**Table 3:** Summary of required computation times and reconstruction deviation metrics for the three benchmarks, providing information about speed and accuracy of the presented method.

**Metric**	**1D**	**2D**	**3D**
Fourier base fitting time	1.18 ms	49.5 ms	144'000 ms
Reconstruction time	0.19 ms	24.3 ms	1070 ms
Max. deviation in mask	0.003	0.05	0.04
Std. deviation in mask	0.002	0.01	0.007
Max. deviation outside mask	0.04	0.09	0.05
Std. deviation outside mask	0.01	0.04	0.01

The reconstruction deviations can be interpreted in comparison to the value range of 1.

#### 3.1.2 2D benchmark

**Figure 4:**
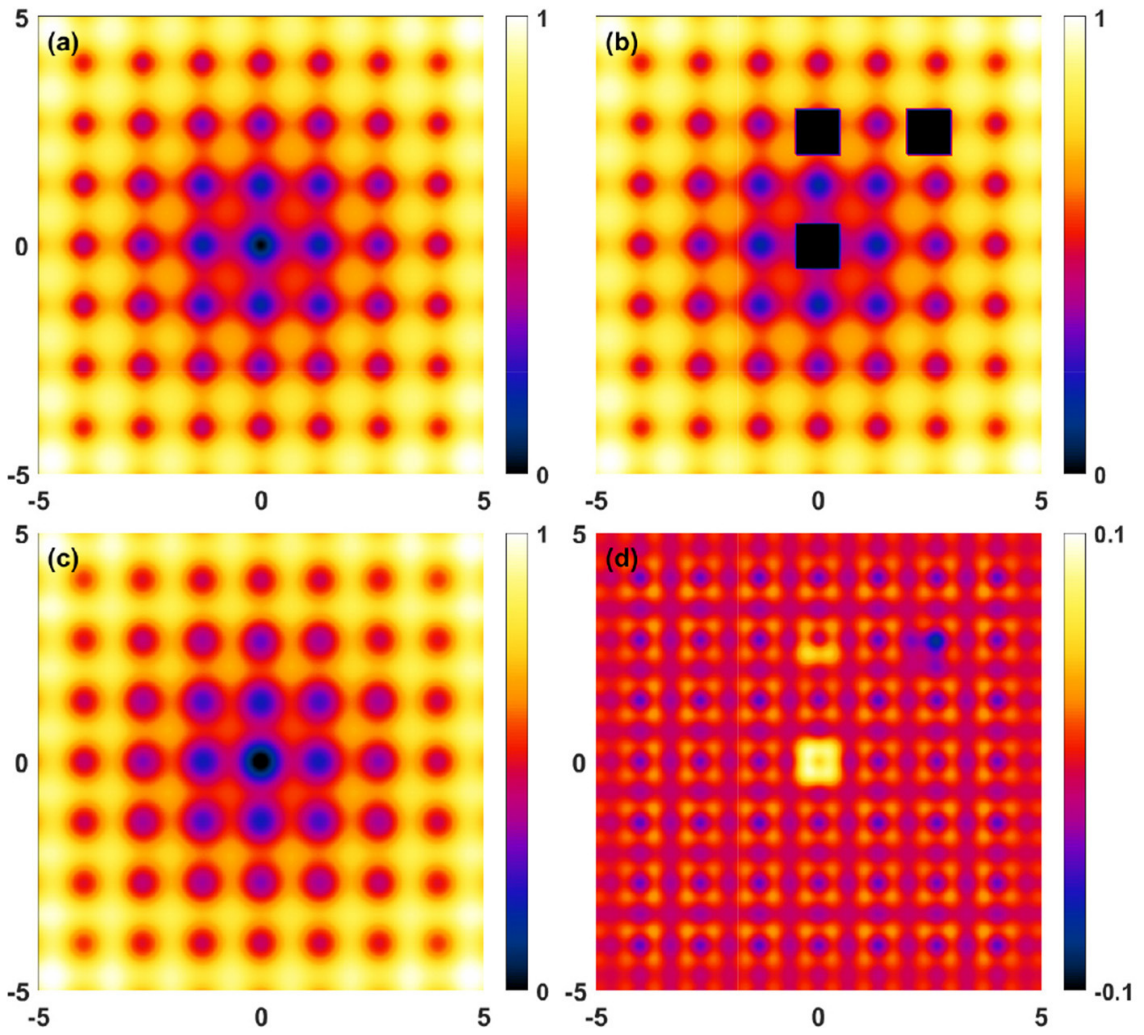
Reconstruction deviations for the 2D benchmark (Equation 14 with *a* = 5, *b* = 0.2, *c* = 1.5π). **(a)** Target function, **(b)** Masked raw data, **(c)** Fourier reconstructed (*N*_*x*_ = *N*_*y*_ = 11) data, **(d)** Reconstruction deviations. Note the different scale in **(d)**.

**Figure 5:**
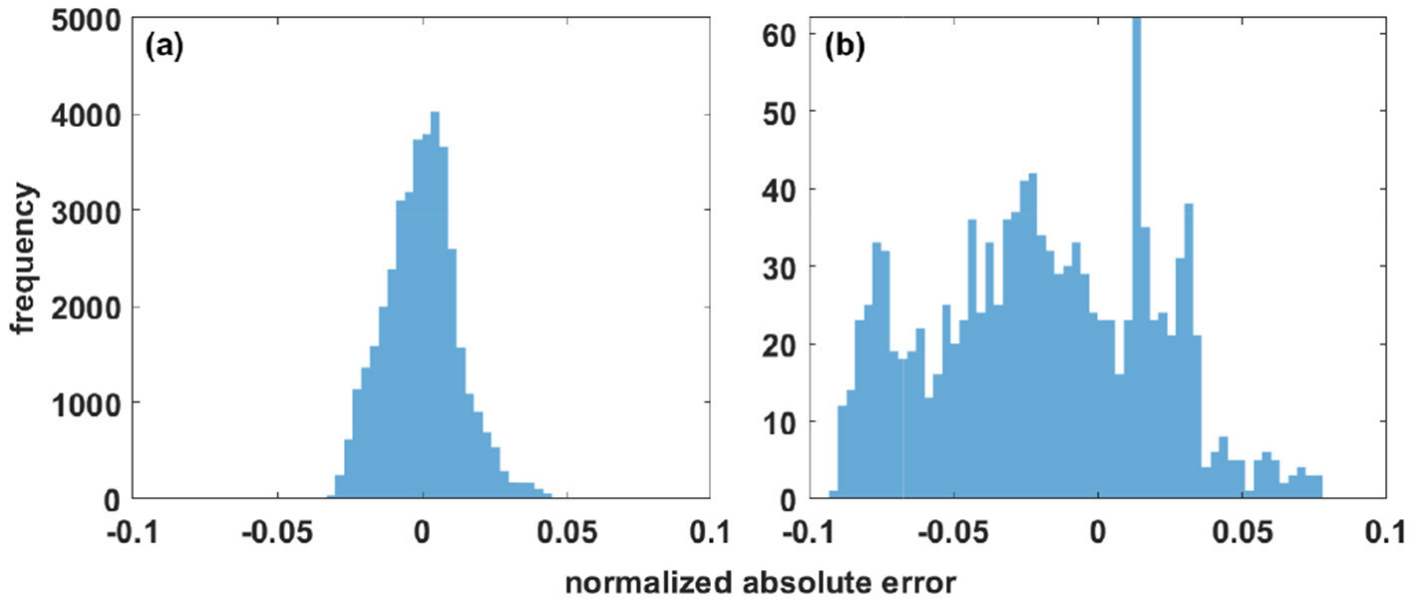
Normalized absolute difference between raw data and reconstructed data for the 2D benchmark presented in Equation 14, expressed in terms of oscillation range (i.e., 1). **(a)** Error within the mask. **(b)** Error within the holes.

**Figure 6:**
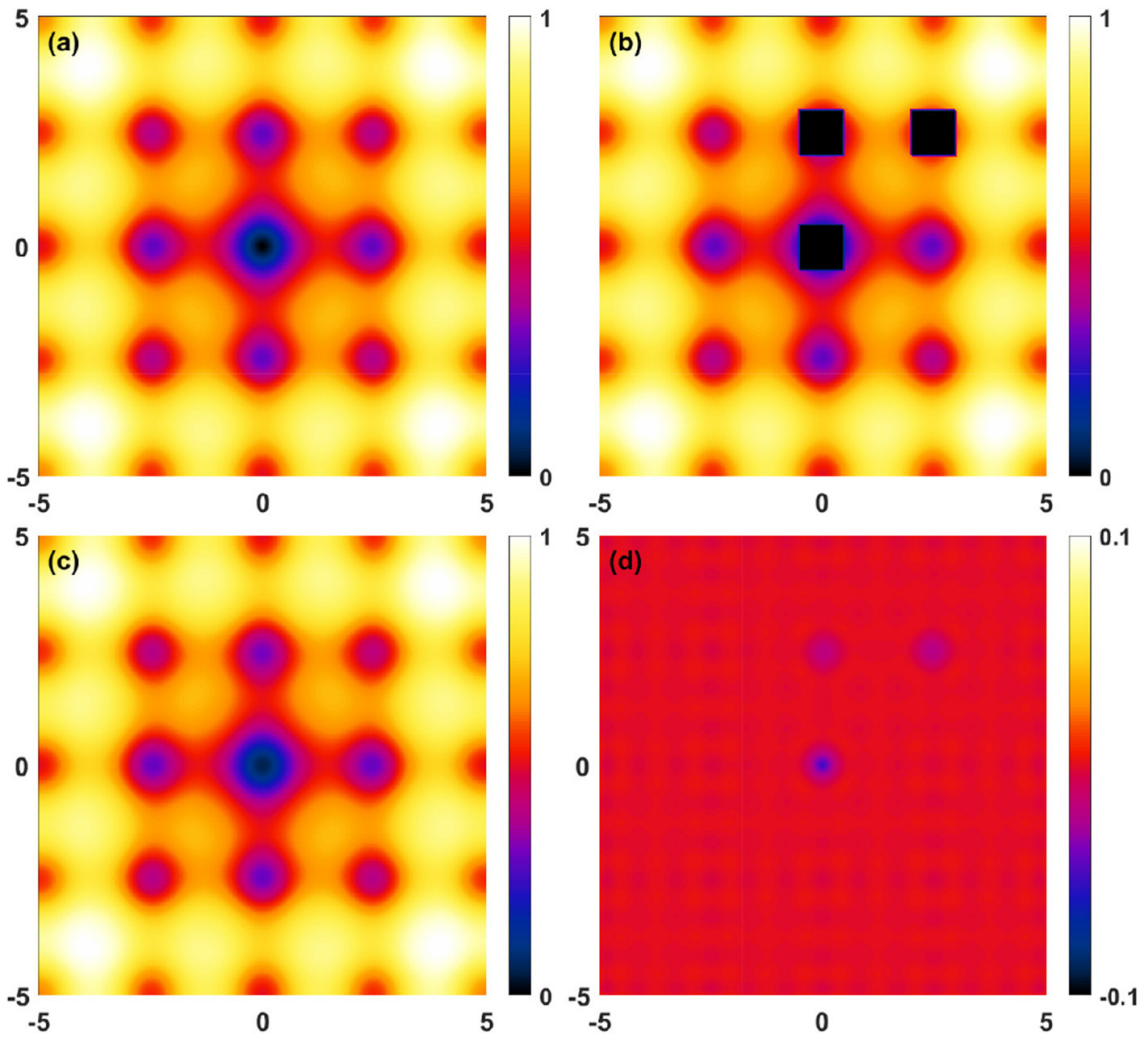
Reconstruction deviations for the 2D benchmark (Equation 14 with *a* = 5, *b* = 0.2, *c* = 0.8π). **(a)** Target function, **(b)** masked raw data, **(c)** Fourier reconstructed (*N*_*x*_ = *N*_*y*_ = 11) data, **(d)** reconstruction deviations. Note the different scale in this figure **(d)**. In this case, the maximal absolute deviations were 0.006 and 0.053 within the masked domain and outside (“holes”), respectively, while the corresponding standard deviations were 0.001 and 0.009. The total value range is 1.

Let *f*_2*D*_(*x, y*) be defined as follows:


(14) $$\begin{array}{l} f_{2D}\left(x,y\right)=-a\exp\left(-b\sqrt{\frac{x^{2}+y^{2}}{2}}\right)\\ \;-\exp\left(\frac{\cos\left(cx\right)+\cos\left(cy\right)}{2}\right)\\ \;+a+\exp\left(1\right) \end{array}$$

on *x, y*:[−5, 5] × [−5, 5]. The interval is discretized into 200 × 200 points and it is assumed that no data is available for *x, y*:[−0.5, −0.5] × [−0.5, 0.5]∪[−0.5, 0.5] × [2.0, 3.0]∪[2.0, 3.0] × [2.0, 3.0]. The reconstruction results (parameters: *a* = 5, *b* = 0.2, *c* = 1.5π, *N*_*x*_ = *N*_*y*_ = 11) are shown in Figure 4. Figure 5 displays the histogram of the reconstruction error, normalized to the oscillation magnitude of the function, for the domains inside and outside the mask. Figure 6 shows corresponding results obtained using slower oscillations (*c* = 0.8π).

#### 3.1.3 3D benchmark

**Figure 7:**
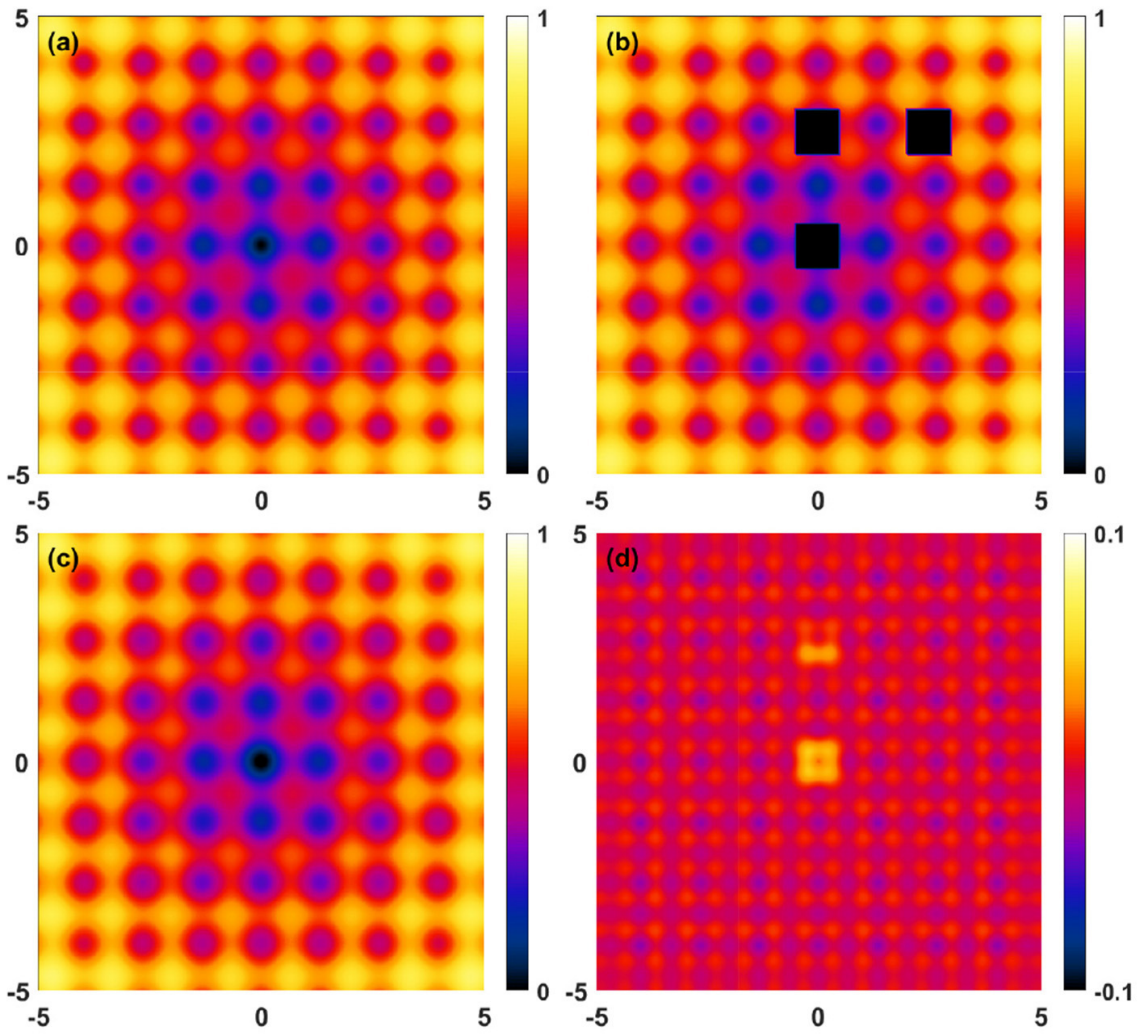
Reconstruction deviations for the 3D benchmark (Equation 15 with *a* = 5, *b* = 0.2, *c* = 1.5π). **(a)** Target function, **(b)** masked raw data, **(c)** Fourier reconstructed (*N*_*x*_ = *N*_*y*_ = *N*_*z*_ = 11) data, **(d)** reconstruction deviations. All results are shown on the *z* = 0 plane.

**Figure 8:**
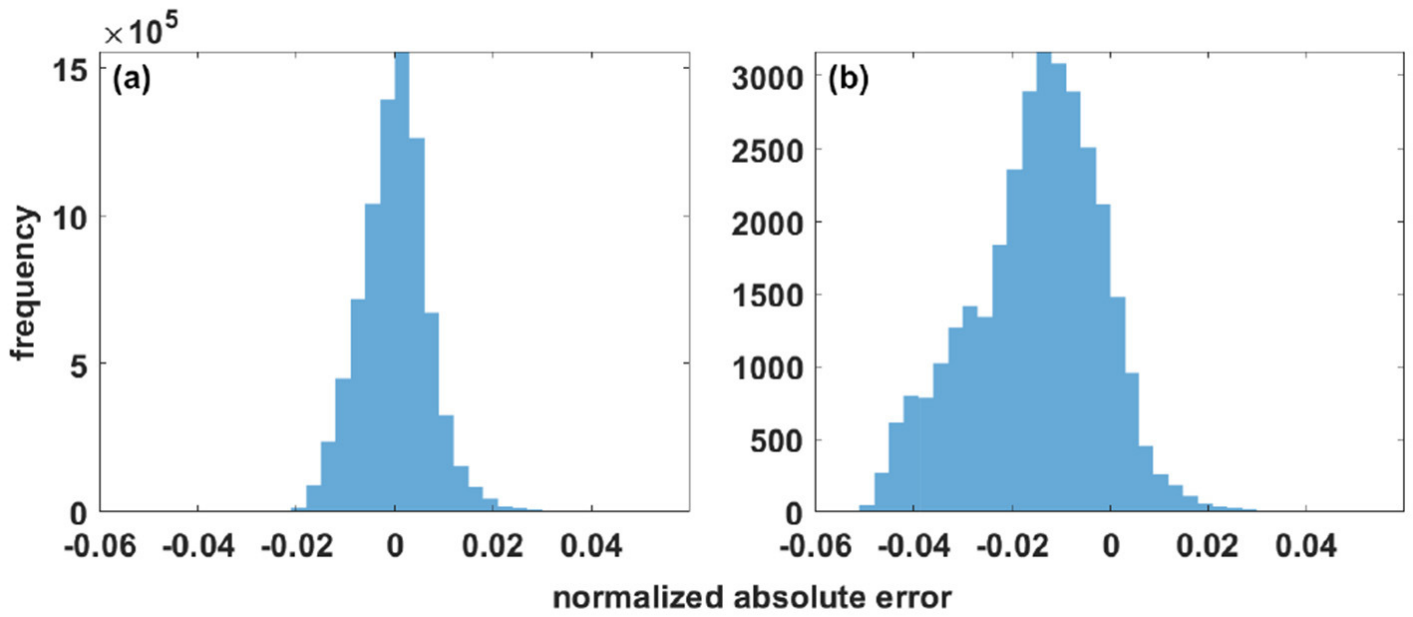
Normalized absolute difference between raw data and reconstructed data for the 3D benchmark presented in Equation 15, expressed in terms of oscillation range (i.e., 1). **(a)** Error within the mask. **(b)** Error within the holes.

Let *f*_3*D*_(*x, y, z*) be defined as follows:


(15) $$\begin{array}{l} f_{3D}\left(x,y,z\right)=-a\exp\left(-b\sqrt{\frac{x^{2}+y^{2}+z^{2}}{3}}\right)\\ \;-\exp\left(\frac{\cos\left(cx\right)+\cos\left(cy\right)+\cos\left(cz\right)}{3}\right)\\ \;+a+\exp\left(1\right) \end{array}$$

on *x, y, z*:[−5, 5] × [−5, 5] × [−5, 5]. The interval is discretized into 200 × 200 × 200 points and it is assumed that no data are available for *x, y, z*:[−0.5, −0.5] × [−0.5, 0.5] × [−0.5, 0.5]∪[−0.5, 0.5] × [2.0, 3.0] × [−0.5, 0.5]∪[2, 3] × [2, 3] × [−0.5, 0.5]∪[−0.5, 0.5] × [−0.5, 0.5] × [2.0, 3.0]. The reconstruction results (parameters: *a* = 5, *b* = 0.2, *c* = 1.5π, *N*_*x*_ = *N*_*y*_ = *N*_*z*_ = 11) are shown in Figure 7. Figure 8 displays the histogram of the reconstruction error, for the domains inside and outside the mask.

### 3.2 Application to deformation data

Deformation imaging reveals how the brain pulsates over a cardiac cycle due to transfer of blood and CSF between cranial and spinal compartments, which affects anatomical geometry and dielectric tissue properties, modulating the head impedance. In consequence, measurement of head impedance modulation can potentially be used to derive non-invasive CC surrogates. Deformation imaging reveals how the brain pulsates over a cardiac cycle due to transfer of blood and CSF between cranial and spinal compartments, which affects anatomical geometry and dielectric tissue properties, modulating the head impedance. In consequence, measurement of head impedance modulation can potentially be used to derive non-invasive CC surrogates. Experimental and modeling work by Spiegelberg et al. (2022) confirmed that head impedance variations can be measured and are at least partially of intracranial origin. A follow-up study Boraschi et al. (2023) demonstrated posture-dependent changes in the non-invasively measured intracranial pulse waveform, providing further evidence that head impedance contains physiologically relevant information. These findings provide strong support for the feasibility of deriving meaningful, biophysically grounded surrogates of CC from surface electrode measurements. To translate this concept into a quantitative method, a computational biophysical model was developed Karimi et al. (2023) that relates small intracranial displacements and dielectric property changes to features of transient multi-contact head impedance variations. The computational model combines brain surface displacement fields over the cardiac cycle with electromagnetic simulations to derive resulting transient impedance variations, before deriving relationships between pulsation characteristics and the measurable signal—hence the necessity to extract surface motion fields from volumetric image data. However, deformation imaging is notoriously unreliable at the brain-CSF interface due to motion artifacts, dielectric-contrast-related noise, and flow-related imaging distortions. To improve the low signal-to-noise ratio of deformation data and replace unreliable data near brain-CSF interfaces, first masking is applied. Masking excludes data outside the brain and within 3 mm of its borders (cortical surface and interface to ventricles). Subsequently, the Fourier reconstruction method is employed for smoothing and extrapolation-based reconstruction.

**Figure 9:**
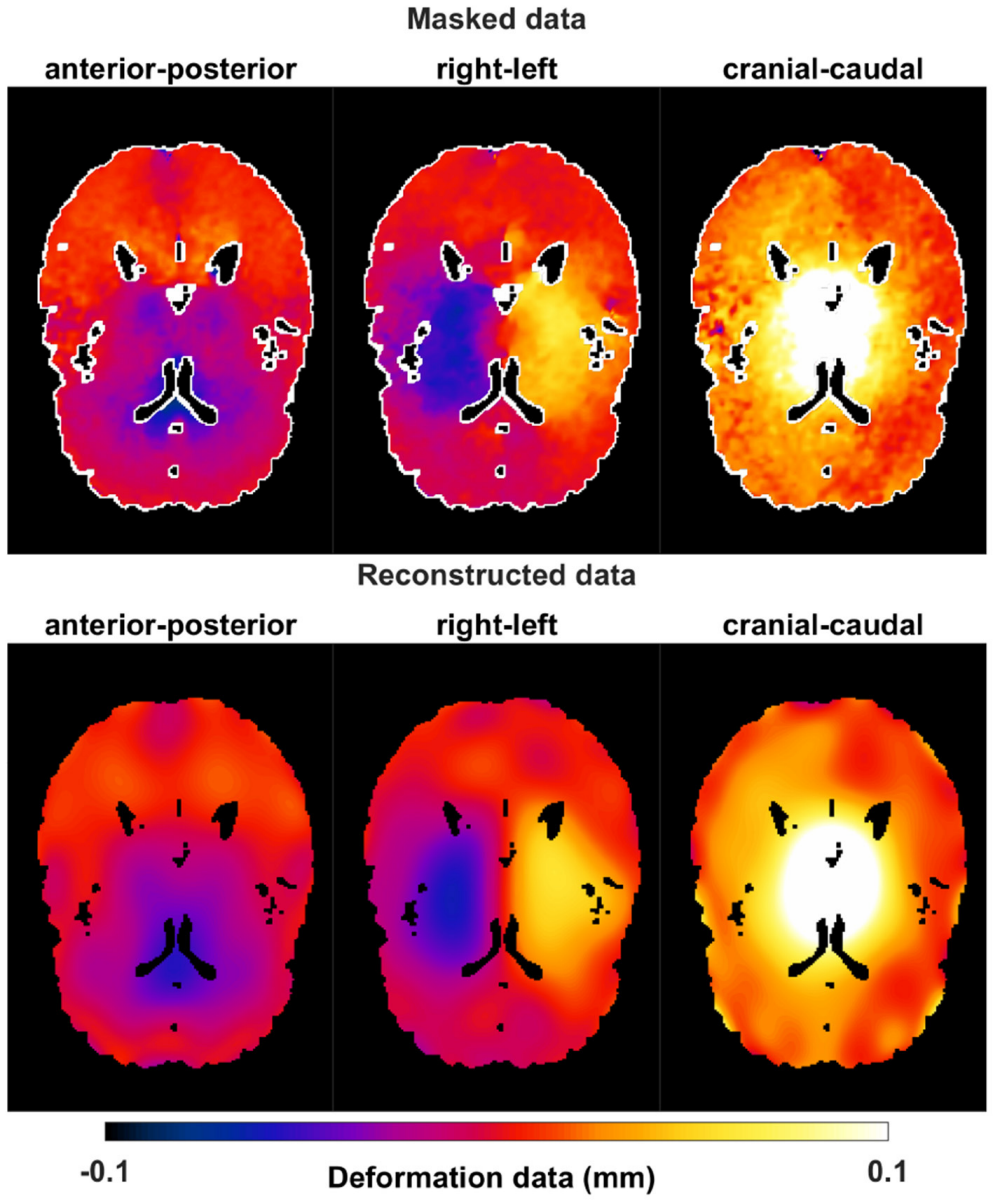
Masked deformation data and reconstructed one using Fourier base fitting (with *N*_*x*_ = *N*_*y*_ = *N*_*z*_ = 4) for one time step and one slice in the brain for right-left, anterior-posterior, and cranial-caudal components. The white parts in the masked data are CSF close-by regions which need to be extrapolated.

The deformation data were acquired using a 7T MR scanner and the DENSE method (Adams et al., 2020), for right-left, anterior-posterior, and cranial-caudal gradient orientations (two opposite gradient polarities were recorded per direction). The resulting 4D data were large, comprising 320*190*320 pixels, 20 snapshots over the cardiac cycle, and 6 polarities. Background noise was reduced by subtracting the data from two opposite polarities. The Fourier basis fitting approach was applied for each time step during the cardiac cycle and each direction (right-left, anterior-posterior, and cranial-caudal). Figure 9 shows masked original deformation data and the smoothed and reconstructed one (setting *N*_*x*_ = *N*_*y*_ = *N*_*z*_ = 4) for one illustrative time step and brain slice.

Fitting the Fourier basis took 1.9*s* for computing the [*A*] matrix (see Equation 8) and its SVD, which needs to be performed only once, regardless of how many imaging time steps and movement components need to be reconstructed. Computing the [*B*] matrix and solving Equation 8 (i.e., base fitting) and reconstruction each took 0.5*s* per time step and directional component.

Importantly, image-based motion reconstruction is only required during the development and validation of the CC monitoring approach—once the required relationships have been established, clinical applications of the non-invasive method will no longer require personalized deformation imaging, which is not readily available (certainly not in continuous monitoring scenarios). Instead, readily accessible head impedance recordings will be sufficient for measuring CC in individual patients.

## 4 Discussion

### 4.1 Complexity and timing

#### 4.1.1 Exponential function evaluation

By precomputing the exponential functions, we reduce the number of corresponding evaluations from (4*N*_*x*_ + 1)·(4*N*_*y*_ + 1)·(4*N*_*z*_ + 1)·*L*_*x*_·*L*_*y*_·*L*_*z*_ + (2*N*_*x*_ + 1)·(2*N*_*y*_ + 1)·(2*N*_*z*_ + 1)·*L*_*x*_·*L*_*y*_·*L*_*z*_ to (4*N*_*x*_ + 1)·*L*_*x*_ + (4*N*_*y*_ + 1)·*L*_*y*_ + (4*N*_*z*_ + 1)·*L*_*z*_. The required number is further reduced if some of the axes have equal length. In addition, the operations can be efficiently vectorized in NumPy, involving the exponential of three outer products. Without precomputing the exponential functions, the matrix assembly time in our implementation increases by about 50 %.

#### 4.1.2 Matrix computation

The calculations in Equation 10 involve *L*_*x*_·*L*_*y*_·(4*N*_*z*_ + 1) + *L*_*x*_·(4*N*_*y*_ + 1)·(4*N*_*z*_ + 1) + (4*N*_*x*_ + 1)·(4*N*_*y*_ + 1)·(4*N*_*z*_ + 1)scalar products, which is already an important complexity improvement when compared to the *L*_*x*_·*L*_*y*_·*L*_*z*_·(4*N*_*x*_ + 1)·(4*N*_*y*_ + 1)·(4*N*_*z*_ + 1) complexity of a naive implementation. In addition, the *L*_*x*_·*L*_*y*_ scalar products used to compute *M*_*z*_(*x, y*) involve the same vector $\left[e^{jk_{z_{i}}z_{1D}}\right]$ and *N*_*x*_ of the scalar products have the vector $\left[e^{jk_{y_{i}}y_{1D}}\right]$ in common. Thanks to current CPU architectures and advanced compiler logics, these operations can be efficiently vectorized and parallelized as 4*N*_*z*_ + 1, 4*N*_*y*_ + 1, and 4*N*_*x*_ + 1 tensor product operations (*tensordot* from NumPy) that each performs a reduction along a spatial direction of *L*_*x*_·*L*_*y*_·*L*_*z*_, *L*_*x*_·*L*_*y*_·(4*N*_*z*_ + 1), respectively *L*_*x*_·(4*N*_*y*_ + 1)·(4*N*_*z*_ + 1)-sized arrays. Similar considerations also apply to Equation 11.

#### 4.1.3 Solving

We resort to SVD to ensure robust operation even when the matrix conditioning prevents direct solving. SVD complexity scales with the third order of the matrix dimension, i.e., ${\left(\left(2N_{x}+1\right)\left(2N_{y}+1\right)\left(2N_{z}+1\right)\right)}^{3}$. Note, however, that this only depends on the number of considered Fourier base vectors, which is typically much smaller than the number of points in the spatial domain. As mentioned earlier, SVD only needs to be performed once, regardless of how many imaging time steps and movement components need to be reconstructed.

#### 4.1.4 Repeated application

As mentioned earlier, it is only necessary to compute *S* and [*AA*], and to perform the SVD and matrix inversion once for a dataset with a constant mask, even if there are multiple time-steps, deformation vector components, etc.

#### 4.1.5 Performance and vectorization

**Figure 10:**
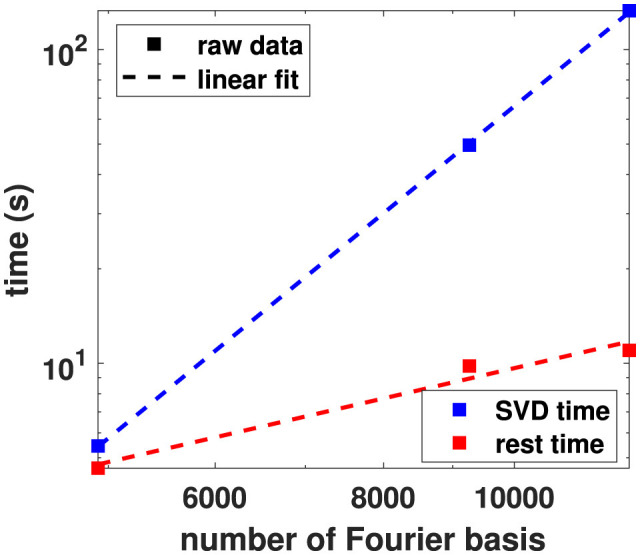
The time required for computing the SVD and performing the other parts of the Fourier fitting procedure for the 3D benchmarks. The x-axis represents the number of Fourier modes ((2*N*_*x*_ + 1)(2*N*_*y*_ + 1)(2*N*_*z*_ + 1)). The data is presented in a log-log plot together with linear regression lines whose slopes reveal the complexity orders of the implemented methods.

The dimension dependence of the benchmark timings presented in Table 3 reflects the increase in the number of points from 200 to 200^3^ (in our specific case where *L*_*x*_ = *L*_*y*_ = *L*_*z*_ = 200) and in the number of Fourier base vectors from 23 (2*N*_*x*_ + 1, with *N*_*x*_ = 11) to 23^3^. The latter aspect quickly dominates algorithmic performance, since the complexity of SVD is $O\left(n^{3}\right)$, when *n* represents the matrix dimension. Indeed, comparison of the 2D and 3D benchmark timings reveals an exponent of 2.5, which is slightly lower than 3.0 (likely because of the non-negligible contribution of better scaling algorithm components and of computational overhead). A closer examination of timings revealed that the SVD operation (implemented using JAX NumPy's linalg svd) in the 3D benchmark accounts for over 92 % (133 s) of the computation time. The remaining 8 % (11 s) is spent precomputing the exponential functions and calculating the various matrix and vector elements of the linear system. Without vectorization, the duration of this part increases to a dominant 843 s, which represents a 75-fold slowdown. The significantly reduced run-time of the deformation data processing, compared to the 3D benchmark—despite the increased number of points—is due to the reduced number of considered Fourier modes (*N*_*i*_ = 4). When varying the number of Fourier modes in the 3D benchmark from (2·11 + 1)^3^ = 12167 to (2·10 + 1)^3^ = 9261 and (2·8 + 1)^3^ = 4913, a scaling exponent of 3.5 (*R*^2^ = 99%) is found for the SVD part and a scaling exponent of 1.0 for the rest (mostly matrix/vector element computations), in close agreement with the theoretical complexities of 3 and 1 (see Figure 10). After compensating the SVD and matrix assembly timings of the benchmarks for their expected scaling as a function of the number of points and Fourier modes—assuming unsuccessful vectorization—we obtain strongly negative regression slopes (−1.3 as a function of the number of Fourier modes, -0.8 as a function of the number of spatial points). These values are a testimony to the gain offered by vectorization.

### 4.2 Choice of *N* and padding

The choice of *N*_*x*_, *N*_*y*_, and *N*_*z*_ is application-specific. Increasing these values reduces smoothing, increases fitting fidelity (higher spacial frequencies are captured), and permits more accurate extrapolation, (albeit generally over shorter distances; interpolation is typically more robust). On the other hand, using fewer modes enhances robustness to noise through low-pass filtering and reduces computational cost. In other words, cut-off selection involves a trade-off between robustness, noise suppression, and computational efficiency versus achievable resolution and fidelity, and is often guided by knowledge about relevant physiological and/or anatomical length scales, e.g., in clinical applications.

As expected, the periodicity of the Fourier basis results in fitted functions that reflect that periodicity. When performing extrapolation, it is therefore important to insert sufficient padding to avoid overly constraining the extrapolation by forcing it to fit a periodic boundary condition. The padding in turn increases the length of the spatial Fourier basis oscillations relative to the extent of the data, such that large padding can necessitate an increase of *N*_*i*_. In the case of the deformation data processed here, a grid extent increase by 10% was found to yield satisfactory results.

### 4.3 Robustness to different missing data patterns

In practical applications, data incompleteness may arise in various forms—ranging from random dropout, to spatially structured missingness, or large contiguous gaps. The developed method is capable of handling all of these, as long as the characteristic length-scale of relevant features is not smaller than the gap size. If critical high-frequency content is absent—due to large contiguous gaps –, accurate reconstruction becomes fundamentally unattainable. This limitation is not unique to our method and can only be overcome if additional prior knowledge is available. Therefore, the size and spatial distribution of the missing regions, as well as the expected spectral content of the underlying data, must be considered before applying this method. The maximum tolerable block size before significant degradation occurs is application-specific and depends on both the frequency characteristics of the data and the desired level of reconstruction detail or smoothness.

### 4.4 Limitations

While the proposed method is robust and efficient for structured grids with arbitrary masking, the following main limitations should be noted. First, the method assumes that the underlying distribution can be well represented by a truncated Fourier basis. This acts as an inherent low-pass filter, which is beneficial for denoising but may limit the ability to reconstruct high-frequency features or sharp discontinuities when they are genuinely present in the data. Second, the optional SVD-based regularization step, while effective in stabilizing ill-conditioned systems, becomes computationally demanding as the number of Fourier modes increases, and may present a bottleneck for large-scale applications. It is also important to note that the method is designed specifically for structured (voxel-based) data, where acceleration strategies such as vectorization and precomputation are most effective. Generalizing it to unstructured grids would require a reformulation of the matrix construction and solution process and is not within the current scope of this work.

## 5 Conclusion

When fitting a Fourier basis to structured data known or defined on a subset of the grid points (mask), a minimization problem must be solved, the Fourier basis loses its unitary property, and determination of the Fourier coefficients becomes non-trivial and computationally expensive. To address this, we developed a library that reduces the number of required operations, minimizes the evaluation of costly non-linear functions, and permits the exploitation of computationally efficient vectorization of vector and matrix operations while reducing the memory footprint. SVD can optionally be employed as part of the solving step to provide regularization when needed—as is often the case. However, SVD quickly becomes the performance limiting factor, as the number of considered Fourier modes increases. The developed method was verified using Ackley function benchmarks and demonstrated in a deformation image processing application. The library has been made publicly available.

## Data Availability

Implementation of the library is available on GitHub, providing open access to the source code. The repository includes documentation to guide users in installing, configuring, and utilizing the library effectively. Researchers and developers can access the library at https://github.com/ITISFoundation/MIFT. Additionally, real deformation data has been published at https://doi.org/10.5281/zenodo.10590047, providing data for utilizing the library in real-world applications.
